# Nourishing Beginnings: A Community-Based Participatory Research Approach to Food Security and Healthy Diets for the “Forgotten” Pre-School Children in South Africa

**DOI:** 10.3390/ijerph22060958

**Published:** 2025-06-18

**Authors:** Gamuchirai Chakona

**Affiliations:** Community Engagement Division, Rhodes University, Makhanda/Grahamstown 6140, South Africa; matarutseg@yahoo.com

**Keywords:** early childhood development, food security, child nutrition, community-based participatory research, dietary diversity, parental engagement, local context

## Abstract

Adequate and diverse diets are essential for children’s physical and cognitive development, yet food insecurity and malnutrition continue to threaten this fundamental right, which remains a pressing concern in many resource-poor settings. This study investigated food and nutrition security in Early Childhood Development (ECD) centres in Makhanda, South Africa, through a community-based participatory research approach. Using a mixed-methods approach combining questionnaire interviews, focus group discussions, direct observations, and community asset mapping across eight ECD centres enrolling 307 children aged 0–5 years, the study engaged ECD facilitators and analysed dietary practices across these centres. Results indicated that financial constraints severely affect the quality and diversity of food provided at the centres, thus undermining the ability to provide nutritionally adequate meals. The average amount spent on food per child per month at the centres was R90 ± R25 (South African Rand). Although three meals were generally offered daily, cost-driven dietary substitutions with cheaper, less diverse alternatives, often at the expense of nutritional value, were common. Despite guidance from Department of Health dieticians, financial limitations contributed to suboptimal feeding practices, with diets dominated by grains and starchy foods, with limited access to and rare consumption of protein-rich foods, dairy, and vitamin A-rich fruits and vegetables. ECD facilitators noted insufficient parental contributions and low engagement in supporting centre operations and child nutrition provision, indicating a gap in awareness and limited nutrition knowledge regarding optimal infant and young child feeding (IYCF) practices. The findings emphasise the need for sustainable, multi-level and community-led interventions, including food gardening, creating ECD centre food banks, parental nutrition education programmes, and enhanced financial literacy among ECD facilitators. Strengthening local food systems and establishing collaborative partnerships with communities and policymakers are essential to improve the nutritional environment in ECD settings. Similarly, enhanced government support mechanisms and policy-level reforms are critical to ensure that children in resource-poor areas receive adequate nutrition. Future research should focus on scalable, locally anchored models for sustainable child nutrition interventions that are contextually grounded, community-driven, and should strengthen the resilience of ECD centres in South Africa.

## 1. Introduction

Good nutrition allows children to survive, grow, develop, learn, play, participate, and contribute, while malnutrition robs them of their futures and leaves young lives hanging in the balance. Stunting is the most devastating form of malnutrition affecting children, as it limits them from attaining their full possible height and impairs the full development of their cognitive potential [1]. Nutritious foods and diverse diets which are of good quality and sufficient quantity are essential for children to meet their nutrient needs and support growth, especially during the first 1000 days of a child’s life (from conception to two years), which are critical for the optimal child growth, health, and development [2]. Draper et al. [3] has also highlighted the importance of the next 1000 days of a child’s life (from 2 to 5 years of age, which include preschool and pre-primary years) in offering a window of opportunity to promote nurturing and caring environments, establishing healthy behaviours, and building on early gains to sustain or improve trajectories of healthy development. Sadly, according to the 2018 Global Nutrition Report [4], children under five years of age face multiple burdens, with 150.8 million stunted, 50.5 million wasted, and childhood overweight and obesity affecting at least 38 million children globally. Within the same report, South Africa is reported as one of the countries with a triple burden of malnutrition, where there are high rates of childhood stunting, anaemia, and overweight in adult women. Additionally, the 2022 Global Nutrition Report [5] has highlighted that the country has shown no progress towards achieving the targets of ending stunting, with 21.4% of children under 5 years of age affected, although it is lower than 27% reported by UNICEF South Africa Nutrition Brief [6] and the average for the Africa region (30.7%). However, other research in the country has reported the highest stunting rates, with one in four children (25%) facing stunted growth due to malnutrition, which is one of the world’s worst rates [7,8]. The prevalence of overweight in children under 5 years of age is 11.6% in South Africa and the country is regarded as being on track to prevent the figure from increasing. However, there is limited progress towards achieving the diet-related non-communicable diseases targets. Poor nutritional quality diets, and inappropriate early child feeding practices exacerbated by a greater access to processed foods [9], among other complex factors such as inadequate sanitation, are contributing to life-threatening infant and child health problems in South Africa.

The South African National Department of Health (NDoH), in its 2013–2017 Roadmap for Nutrition [10], highlighted that the country is undergoing a nutrition transition which is posing complex challenges in the country. This is characterised by the coexistence of undernutrition (notably stunting and micronutrient deficiencies) alongside a rising incidence of overweight and obesity, and related non-communicable diseases such as hypertension, cardiovascular disease, and diabetes. Additionally, millions of South African households struggle to afford nutritious foods, and what is accessible often lacks the essential nutrients needed for healthy growth and development [11]. Household poverty, mainly due to high unemployment translates to food insecurity and is greatly affecting young children’s nutritional status. About 19.7% of the households in South Africa have experienced moderate to severe food insecurity in 2023, while 8% experienced severe food insecurity [11], with approximately 70% of the children living in households relying on at least one social grant [12,13]. Statistics South Africa [14] has highlighted that about 39.4% of households and persons benefit from the social grants and that these are the second most important source of income for nearly 51% households in the country. According to the 2013 South African National Health and Nutrition Examination Survey (SANHANES-1) [15] and the National Department of Health et al. [16]’s South Africa Demographic and Health Survey (SADHS) 2016, the rates of stunting and wasting in children under five years of age have been continuously increasing over the years. Both studies noted that children often skip meals or do not eat all day due to limited access to food, mostly due to the challenges of affordability caused by poverty, unemployment, and being in a single-parent household. Sadly, when these children eat, they have limited food types, which limits their dietary diversity and thus exposes them to hidden hunger [17].

South Africa has policies for and a commitment to improve infant and child health and nutrition as well as malnutrition in infants and young children. One of the guiding principles in the 2013 Infants and Young Children Feeding (IYCF) policy is to promote healthy eating amongst infants and young children and encourage feeding practices that are unharmful to them [18]. However, the focus on the next 1000 days is overlooked, even globally, which has dire implications on the child’s life [3]. For example, policies to improve health and nutrition for children in South Africa often neglect the age group of 2–6 years, which includes children attending the Early Childhood Development (ECD) centres. Complementary feeding of infant and young children is less prioritised and under-resourced [19], with limited data, even in the national food consumption surveys, for this age group. Sadly, the global and regional child malnutrition estimates produced jointly by UNICEF, WHO, and World Bank [1] revealed that the world is still far from a world without malnutrition, especially in South Africa where undernutrition and overnutrition remains a dual nutritional pandemic [5,18].

Early Childhood Development (ECD) centres, defined by the South African government as partial care facilities, provide an early childhood programme which focuses on early learning and development of children from 0 to 6 years [20]. According to the National Integrated Early Childhood Development (NIECD) Policy 2015 [21], the ECD centres have an essential package to promote children’s development which has six cross-cutting components that are considered essential for vulnerable children. These include nutritional support, maternal health care, child health care, social services, support for primary caregivers, and stimulation for early learning programmes to both mothers/caregivers and children [21]. At the ECD centres are facilitators who play an important role as educators, caregivers, and advocates, providing essential support for the holistic growth and development of young children, especially in the crucial formative years (0–5 years). Therefore, ECD centres, together with clinics and hospitals, offer primary health care and are considered significant delivery platforms for nutritional intervention programmes for young children [22], especially those from households with not enough food and who are forced to skip a number of meals. For example, Stats SA [20] reported that almost 21% of children from 0 to 6 years had skipped a meal in the past 12 months prior to the General Household Survey, with 35.7% skipping a meal in the past 30 days. Some of these children were provided with food once daily, with some receiving food a few times a week or sometimes at the ECD centre they attended.

Therefore, multisectoral coordination and integration across the life course is essential to promote increased dietary diversity and intake, inclusive nutrition support, and create opportunities for early intervention that enable all children to thrive and improve their physical growth, as noted by Draper et al. [3]. Applying a multidisciplinary approach involving different stakeholders including academic researchers, personnel in the fields of agriculture, nutrition and health, education, policymakers, local municipality, and the community at large is very critical in co-designing intervention programmes. Furthermore, promoting dietary diversification through nutrition-sensitive agriculture at ECD centres with emphasis on food production and training in how to care for and keep gardens productive may increase access to diversified nutrient-rich foods, especially essential vitamin A-rich vegetables and fruits. It is also critical to focus on empowerment skills that can improve communities’ and families’ nutrition behaviour. For example, research by Bezner Kerr et al. [23] and Karmacharya et al. [24] have shown the importance of communities and local knowledge in influencing IYCF practices. Additionally, a common understanding amongst stakeholders and strengthening advocacy efforts will promote the co-creation of knowledge and bridging the knowledge gap on the integration of nutrition education and agricultural production, thus making implementation of the strategy most effective in terms of project design, delivery, and enhancing capacities within the community. Another determinant of child health is the promotion of nutrition education for mothers and caregivers of young children (≤5 years) [9,25,26], aimed at enhancing their knowledge, building their capacity, and empowering local communities, including ECD facilitators. Through these interventions, many households and communities may be able to feed themselves and their infants and young children with diversified diets, which has high potential to improve their nutritional status [27,28,29]. Thus, a mix of nutrition education and child-centred nutrition-sensitive agriculture can be a powerful intervention strategy to fight infant and young child malnutrition in South Africa.

According to May et al. [22], a child-centred food system approach, ideally, should underpin all six dimensions of food security, which are availability, access, utilisation, stability, agency, and sustainability. Therefore, nutritious food should be available to children at all times, be physically and economically accessible to all, safely and appropriately utilised, and stable and resilient in times of stress and shock. Additionally, food systems should be empowering, enabling the most marginalised to participate in decision-making, while promoting environmental, social, and economic sustainability. Therefore, there is need for concurrent and well-designed nutrition education and behaviour-change approaches; community empowerment targeting mothers, caregivers, and ECD facilitators; and multidisciplinary/intersectoral collaborations for this to translate into improved diets and nutrition [30]. Additionally, implementation programmes should be people-centred and not take a top-down approach, which should start with the lived experiences/realities of children and their mothers/caregivers and the ECD facilitators, and then use a child-centred assessment to identify necessary actions throughout the food system [13]. As highlighted by Cristina Duarte, the United Nations Under- Secretary-General and Special Adviser on Africa at the United Nations Food Systems Summit in 2023, in Africa, “when you feed a child, you feed the mother, when you feed the mother, you feed the family, when you feed the family, you feed the community…”. It is important to implement contextualised nutrition interventions tailored for specific populations and areas, ensuring they suit the ECD centres and community needs to effectively improve young child feeding practices [19]. Therefore, integrating nutrition and ECD services provide an opportunity to extend continuous support to young children enrolled at ECD centres [31], especially the 2–5-year-olds, who are indirectly considered to be ‘outside’ the priority age range for nutrition interventions. This integration also offers a vital platform to influence caregivers and mothers, whose active involvement is essential for effectively improving children’s nutritional status. 

South Africa continues to face significant food insecurity and nutrition challenges, particularly in the Eastern Cape province, which has the highest prevalence of stunting in young children (24.8%) [32]. In this context, community-based participatory learning and action cycles that promote a mix of nutrition education for mothers and ECD facilitators and a child-centred food system promoting nutrition-sensitive agriculture become significant. This approach has been used successfully with mothers and caregiver groups in other countries [33,34], which has significantly improved maternal-child health outcomes. Thus, maternal and caregiver education have significant impacts on reducing child malnutrition [35,36]. Similarly, having knowledge of appropriate child feeding practices is fundamental for the survival, growth, development, health, and nutrition outcomes of infants and young children [37]. However, these studies focused on the importance of community participation in improving food and nutrition security rather than acknowledging the role of participants in knowledge co-creation and highlighting the beneficial nature of the research to both participants and the communities.

There is limited knowledge in South Africa on the use of community-based participatory research (CBPR) in the ECD space, specifically with focus on nutrition. In this context, CBPR involves the collaboration between community partners, researchers, and other stakeholders to address nutritional issues affecting young children to improve their growth and health outcomes. All collaborators are empowered, as they contribute their expertise and participate in design, decision-making, and ensuring that research is relevant and impactful for the community. Therefore, the present study aimed to assess and improve the food and nutrition security of infants and young children (0–5 years) enrolled in ECD centres in Makhanda, located in the Eastern Cape province of South Africa, using a CBPR approach. This included examining feeding practices and the nutritional status of children, evaluating the sources and adequacy of food provided, identifying socio-economic factors influencing nutrition, and developing context-specific guidance and supportive environments to promote optimal IYCF practices through active engagement with mothers, caregivers, ECD facilitators, and other stakeholders. The research questions were designed to gather information on the food security and dietary intake of infants and young children at the ECD centres, the sources of the food consumed by these children when at the centres, whether it was provided at the centres or packed from home, and whether the food was enough, safe, and nutritious. Questionnaires were used to obtain information on diets and dietary diversity for children at the selected ECD centres and the socio-economic factors that could impact children’s nutrient intake, their growth, health, and development. Through focus group discussions with the caregivers and ECD facilitators, perceptions on IYCF practices in the context of facilitators’ knowledge regarding children’s diets and dietary changes, parental involvement and decision-making on what is consumed at the centres were explored. The study also determined the drivers and barriers to food security at ECD centres, providing some guidance to solutions to combat food insecurity and malnutrition in young children, even in exceptionally difficult circumstances. Lastly, the study created an enabling environment for the implementation of a specific child-centred nutrition-sensitive agricultural project to enhance local capacity development and strengthen networks, and identified potential entry points for intervention and expansion (capacity building and social learning processes) within the Makhanda ECD environment. However, commitment by all stakeholders (specifically the ECD facilitators and caregivers) to intervention programmes that promote optimal feeding practices for the infants and young children, especially those attending ECD was encouraged for the interventions to be successful [33,36]. The study collaborated with different stakeholders in Makhanda to co-create and integrate nutrition education knowledge into child-centred nutrition-sensitive agricultural activities that improves infant and young child health and nutrition.

## 2. Materials and Methods

### 2.1. Study Area

**Figure 1:**
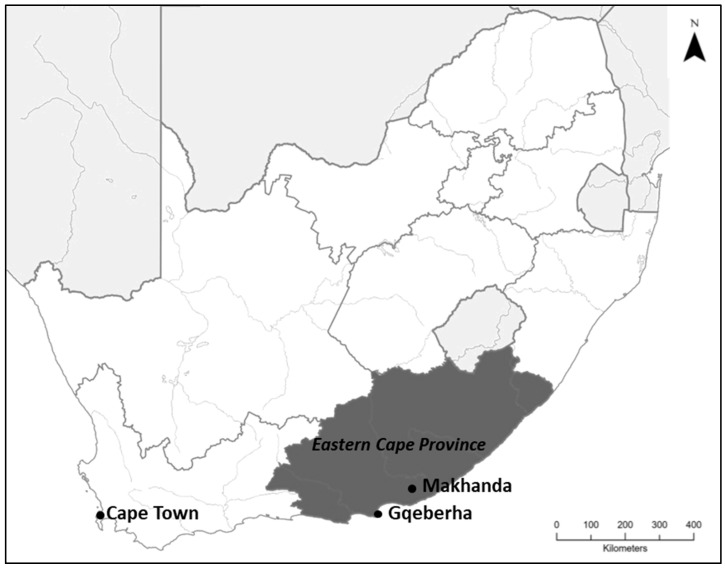
Map of South Africa showing the location of Makhanda in the Eastern Cape Province.

The study was carried out with eight ECD centres in Makhanda which have a community–university partnership with Rhodes University and who had granted their consent to participate in the study (Figure 1).

Makhanda (formerly known as Grahamstown) is the primary location of Rhodes University. This study supported the standing relationships of the ECD centres with Rhodes University Community Engagement and has promoted the nurturing of community–university partnerships that contribute to sustainable community development. The town is situated in the Makana Local Municipality, which is one of the seven local municipalities in the Sarah Baartman District in the Eastern Cape province [38]. Makana Local Municipality covers an area of approximately 4376 km^2^, with a population of 97,815 and approximately 21,388 households [38,39]. Makhanda holds the municipality’s main seat, as 90% of the municipal population live in the town.

The area is characterised by high unemployment rates (17.9%), which greatly affects the youth [38]. Additionally, disruptions from the COVID-19 pandemic and the adverse effects of climate change have decreased food availability for most households, thus worsening the food and nutrition insecurity and economic status of the most vulnerable families in Makhanda [38]. The majority of households do not practise crop production but rely on food purchasing, mostly from income received from the government grants [40], which is similar to most towns in South Africa [9,41]. About 63.2% of people in Makhanda were found to be living in poverty in 2021, and almost 60% are living below the food poverty line [38], with communities in Grahamstown East, who are mostly of African origin, being most affected. The majority of households (at least 53%) are female-headed and the percentage of children within the municipality is about 26.5% [38].

According to the Education Summit held at Rhodes University in January 2024, a study on the state of ECD centres in Makhanda by the Centre for Social Development (CSD) has noted that only 50% of children between 2 and 6 years are enrolled in an ECD service, with the other half staying at home. This is mainly due to the socio-economic challenges faced by parents and caregivers as there are no free ECD services in South Africa. Additionally, of the 71 ECD centres in the CSD database, only 8% of these centres have full registration with the Department of Basic Education (DBE); 47% have received conditional registration, with approximately 25% having an unknown status and 20% not registered due to failure to meet all the requirements needed by the department. Thus, only 55% of the ECD centres in Makhanda receive a conditional grant/subsidy from the DBE which is supposed to be used for administration (including salaries), stimulation (books, puzzles, etc.), and for nutrition. The other 45% raise their income through fees. Although the ECD centres provide food for the children in attendance, nutrition is underfunded and food insecurity is a threat to the young children, thus increasing the rates of stunting in the town.

### 2.2. Sampling Procedure

The study was conducted from May 2024 to February 2025. The study employed multiple methodological approaches to underpin its overall focus, which falls in the nexus of infant and child health, nutrition, education and training, information/knowledge creation, and communication. Overall, the project used a community-based participatory research (CBPR) approach, where the research took place within the ECD communities of Makhanda where community knowledge and voices were acknowledged and included in the research. We used the participatory–collaborative–contextual approach to knowledge co-creation and production, which has the potential to ensure the relevance of the research into the realities of the ECD communities in Makhanda, as well as harnessing community wisdom throughout the research process.

A mixed-methods research approach combining both qualitative and quantitative techniques was used [42]. Quantitative data was collected through questionnaires during survey interviews with the ECD facilitators at the ECD centres and through observations at the ECD centres, while focus group discussions with the facilitators were carried out to collect qualitative data. Focus group discussions and qualitative observations complemented ECD centres’ surveys and these added more insights into collective meanings attached to IYCF practices at the ECD centres in Makhanda, creating important information that could have not been elicited by questioning individual facilitators. With the focus group interactions, we managed to develop an understanding of the meaning and experiences of peoples’ lives (in this case, the ECD facilitators) from the point of view of those who experience it. We conducted all interviews in the respondents’ preferred language of IsiXhosa or English, with well-trained translators and enumerators who had full understanding of the questions administered. We also used both languages for focus group discussions and all other interactions that happened between Rhodes University researchers and the community’s ECD facilitators. The research received Ethics approval from Rhodes University (2023-7069-7470 and 2024-7069-8936) and the Eastern Cape Department of Health (EC_202303_013). The facilitators provided written or oral consent before each interview and the focus group discussions. Sampling was spread throughout the month to cater for the times when ECD centres would have received fee payment from the parents and from the quarterly government subsidy. We had interactions with the ECD centres at least thrice and for some visits, we deliberately visited the centres either between 10h00 and 11h00 or between 12h00 and 13h00 when the children were on food/fruit break and lunch respectively. During these times, researchers were able to observe what the children were consuming.

### 2.3. ECD Centre Surveys

The study used a non-random purposive sampling approach [43], where data was collected using the deliberate choice of ECD centres which have partnership with Rhodes University. We targeted facilitators at the ECD centres who actively participate in activities with Rhodes University Community Engagement, which has cemented the relationship and promoted this community–university partnership for sustainable community development. Eight facilitators at the eight ECD centres located in different proximities in Makhanda were interviewed. The facilitators were asked to name all the food that was consumed by the children when they are at the centre, which included all dishes, snacks, and drinks. They were encouraged to remember all the foods consumed per meal and in-between meals, fully describing all the ingredients in mixed dishes. The surveys included open-ended questions on IYCF practices, specifically on what the facilitators perceived as a good quality diet for children, what they actually feed the children with, how often per day, and the reasons for this. We determined the food group intake for infant and young children up to 24 months of age using the seven-category child indicator, as recommended by the World Health Organisation (WHO) [44]. The seven recommended food groups for children are (1) grains, roots, and tubers; (2) legumes, nuts, and seeds; (3) dairy products; (4) flesh foods (meat, fish, poultry, and liver/organ meats); (5) eggs; (6) vitamin A-rich fruits and vegetables; and (7) other fruits and vegetables. These practices also apply to children from 24 to 60 months, who are expected to consume more foods, approximately three meals per day with two snacks in between, to meet their nutritional needs. This should be done more often, with continuous intake of a variety of foods. The child indicator we followed only represents the complementary foods in the diet, excluding breast milk intake, and children are expected to consume a minimum of four food groups per day out of the seven recommended [44].

### 2.4. Focus Group Discussions

The study also implemented focus group discussions (FGDs) with ECD facilitators, which actively made use of the group interactions on the issues relevant to a specific topic [45,46]. In this case, the focus group discussions gave insights into the collective meanings of IYCF practices at the ECD centres, which could not have been elicited through the questionnaires during surveys. Thus, the study was approached from a fresh and different viewpoint, posing new questions that would lead to innovative directions, leading to solutions to improve IYCF practices. We were able to obtain information on the challenges and concerns on the status of the ECD centres, the IYCF practices and the facilitators’ individual experiences and feelings in this regard, and why they acted in the way they did. Therefore, the FGDs developed an understanding of the meanings and experiences of the children’s lives at the ECD centres from the point of view of those who experience it, the ECD facilitators. All facilitators were female. They were able to express their views and opinions, openly discussing child nutrition, health, and development issues that are largely deemed as parental and/or caregivers’ issues, particularly if you are women. We also managed to obtain information on issues of parental involvement in the day-to-day running of the centres. The FGDs comprised the eight facilitators who had participated in the surveys and we welcomed an additional three from other ECD centres who were interested in joining the discussion after hearing about it from the others.

The FGDs were held in comfortable, peaceful, and convenient settings at Rhodes University and the discussions took between 60 and 90 min. To minimise the challenges that would have existed due to language sensitivity and the researcher lacking relevant identity or direct experience, and not being able to comprehend what would be happening in the communities, a research assistant familiar with the place and its norms was recruited. Both the principal researcher and an assistant made notes and recorded the discussions, only after consent was provided by the ECD facilitators. Reflexivity was a critical issue in this study prior to the FGDs. As the research team, we situated ourselves socially and emotionally in relation to our study participants, who were the ECD facilitators. Following Chakona and Shackleton [26], we positioned ourselves as “strangers in a strange land” who were studying the unfamiliar subjects, thus bringing awareness of unconscious bias to the participants. This was advantageous because the researcher was considered ‘ignorant’ whilst the respondents were held to be in the expert position, thus bringing about a knowledge-imbuing experience. All FGD questions were open-ended, with new questions arising from the responses given, as participants were able to build on each other’s ideas and comments. The FGDs sought responses to the following summary of thematic questions addressing issues on the following topics:

1. Sustainable Funding Solutions: How can ECD centres prepare for government funding delays to ensure food provision and staff payment without relying on additional parental support?
2. Food Gardens: Can growing food at ECD centres help address food shortages? What are the challenges preventing some centres from maintaining gardens, and what alternatives exist for centres without gardening space?
3. Menu Adjustments and Affordability: How can ECD centres work with the Department of Health to develop a nutritious, affordable, and adaptable menu that meets children’s preferences while ensuring food supply stability?
4. Parental Involvement: How can parents be more engaged in their children’s nutrition at ECD centres, especially in contributing food during shortages? What strategies have been successful in involving parents?
5. Healthy Eating Awareness: How can parents be encouraged to provide healthier meals for their children when at home, especially during school holidays, to prevent health decline?
6. Holiday Meal Provision: Is it feasible for ECD centres to provide at least one meal for children during holidays? If so, how could this be implemented?

### 2.5. Researcher-Driven Observations and Community Asset Mapping

During the site visits, the researcher used observations as a data collection technique to understand the feeding practices at the ECD centres. We collected about eight observations of children being fed at the ECD centres. With consent from the ECD facilitators, the researcher managed to record the types of foods that the children were consuming. Observations were also used for understanding the general attendance of the children at the ECD centres. Thus, observations were used in complementarity with questionnaire surveys and focus group discussions as a supplemental means of corroborating research findings and increasing the validity of the study. The researcher desired to gain a deeper understanding of social dynamics and gain an understanding of what was actually being consumed by the children when they are at the ECD centres and also whether all the children in the register were able to attend classes consistently. Additionally, we carried out community asset mapping for each site to determine if there were any resources closer to the ECD centres which could be used for developmental projects for the centres. We specifically looked for churches, clinics, any community gardens, community health workers, and whether there were any active water supply sources, including water tanks.

### 2.6. Data Analysis

Quantitative data were entered and cleaned using Microsoft Excel and statistical analyses were performed using Statistica version 14.1.0.8 (StatSoft Inc., Tulsa, OK, USA). Descriptive data are presented as means and SDs (mean ± SD) and percentages. Additionally, qualitative data from the FGDs, which were mostly hand-written field notes and recording, were entered into Microsoft Word 15 and were edited. All responses in IsiXhosa were translated to English, ensuring that we did not lose the meaning of the conversations. The researcher read these transcripts several times to understand the information. We then used qualitative content analysis to interpret textual data content by using a systematic classification process that involves coding to identify patterns or themes. We followed four steps which included repeated review of the transcript to gain a thorough sense of the overall content in the texts, identifying central meaningful units in the material, condensation of the content through a coding of the text, and finally creating categories that contain the condensed meaning of the main themes in the material [47]. Themes were then analysed through coding [48] using NVivo 1.7.2 (Lumivero (formerly QSR International), Burlington, MA, USA) qualitative data analysis software and similarities between the codes were identified and those that were connected were combined to form primary themes for the discussions. To ensure that the data analysis was a trustworthy representation of the themes from the FGDs rather than the researcher’s biased reflection, the research assistant was constantly consulted to examine the accuracy of the analysis. Most sections of the discussions were quoted verbatim, with a few modifications to increase readability.

## 3. Results

### 3.1. Sample Description

The sample consisted of eight ECD facilitators from the ECD centres situated in Grahamstown East, Makhanda. The total number of children in the register at these ECD centres was 307, with the number of children ranging from 26 to 60 children per ECD centre (Table 1). The mean number of children per centre was 38 ± 11 children, with 52.4% being boys and 47.6% girls. The youngest child at the ECD centres was seven months old and the oldest was 60 months old. The mean age of the youngest child per centre was 16.6 ± 6.3 months old whilst the mean age of the oldest child was 57 ± 4.5 months old. Almost all the ECD centres open at 07:30 hrs in the morning and close at 15:00 hrs in the afternoon, with a few opening at 07:00 or 07:30 and closing at 16h30 and/or 17h00 hrs. Some children spent up to 10 hrs at the centres, although the average number of hours spent by children at the ECD centre was 8.1 ± 1.4 hrs.

On average, the ECD centres spent about R3563 ± R1489 on food per month, with the poorest ECD centre only spending R1300 whilst the highest amount spent on food was R5000 per month, mostly by those ECD centres receiving support from Child Welfare or churches (Table 1). Thus, the mean amount spent per child per month was ZAR 90 ± ZAR 25. The facilitators indicated that the amount for food was not enough, which required major cost-cutting and improvisation. However, from the observations we made, not all the children in the register attended the classes every day. Less than 50% of the total number of the children in the register were at school for all the times we visited the centres. This was mostly due to the parents not having paid the fees for the month and, less likely, the child being sick and staying at home. The facilitators have also reported that some parents were not paying school fees, leading to financial difficulties for the ECD, while others withdrew their children from the ECD centres to avoid paying fees and would wait for Grade R (the first year of schooling before Grade 1) enrolment, which is fee-free.

### 3.2. Food Consumption Patterns and Children’s Diet at the ECD Centres

The food consumption patterns and diets were similar across all the eight ECD centres. Children were provided with three meals at the centre which consisted of breakfast, midmorning snack, and lunch. The food for breakfast and lunch were prepared at the centres, whilst children brought food for the midmorning snack and fruit time from home. The ECD facilitators reported that the food that the children usually bring from home included fruit, mostly apple, orange, and/or banana; yoghurt; bread with peanut butter, jam, margarine, or polony (processed meat); chips (Nik Naks, which are made from corn); noodles; diluted juice; and water. For some of the young children who were under two years, the mothers also packed bottled milk for them. However, not all the children would bring food to school, as noted by the ECD facilitators. In cases like these, the facilitators would provide the children with their own food or share the food equally amongst the children, teaching them ways of kindness using the phrase “sharing is caring”.

All the ECD centres prepared breakfast and lunch at the centres every week day, following the menu and guidance that they were given by dieticians from the Department of Health, which all the facilitators were happy with. The ECD facilitators, their assistants, and those responsible for cooking at the centres attended training on how to prepare the food correctly without destroying nutrients in the food. Thus, they all had extensive knowledge on nutritious foods suitable for the children and the IYCF practices, including prioritising food safety. The foods that the facilitators prepared for the children for breakfast included oats, mealie meal porridge, and Morvite and amabele porridge, which are both made from sorghum. The porridge was sometimes served with fresh milk but mostly with powdered milk, which they mentioned as being Cremora (a coffee creamer).

**Figure 2:**
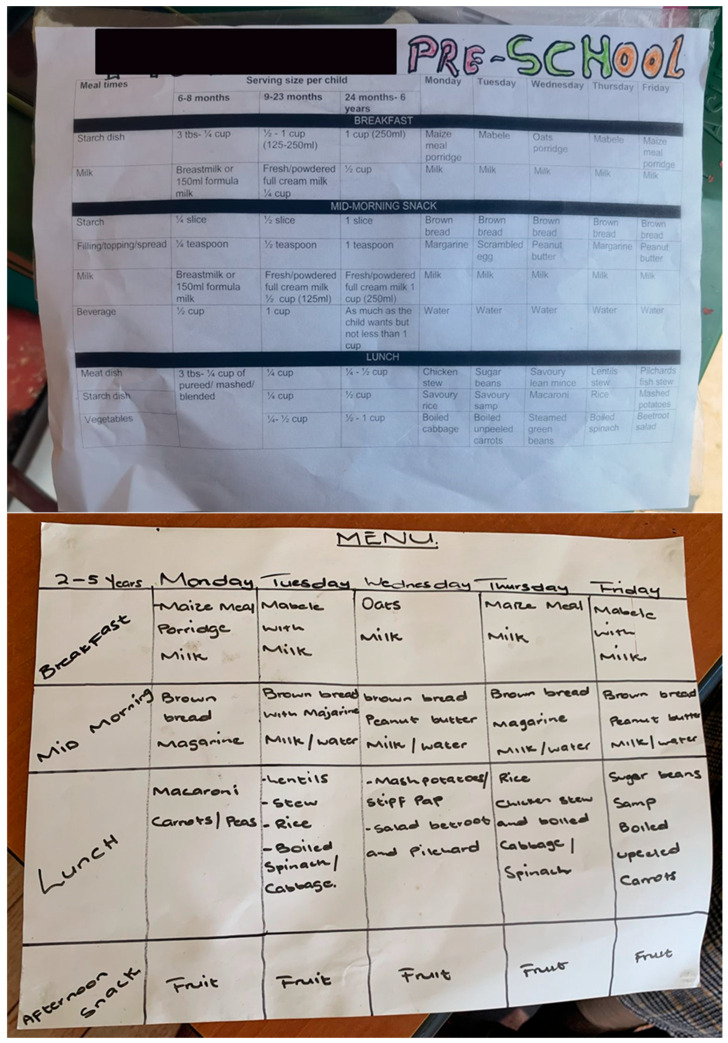
Some examples of the menu used by guiding facilitators to prepare nutritious foods for the children.

Children were given different types of food for lunch; depending on the day, there was variation in what would be on the menu (Figure 2) and what was available. For the group grains, roots, and tubers, they were provided with pap made from mealie meal, macaroni, potatoes, rice, samp (crushed corn), and brown bread, with only one ECD centre making voetkoek for the children. For the group legumes, nuts, and seeds, they received beans, lentils, Imana soya mince soup, and peanut butter, whilst they received maas (fermented milk) and sometimes fresh milk for the dairy products group. Cheese was rarely consumed at the ECD centres, as only 25% of the facilitators reported that some children consumed it only when packed from home. All the facilitators also reported that they prepare flesh foods for the children, which included chicken livers, chicken, Russian sausages, and pilchards (canned fish), with rare occasions when they prepare lean beef mince, as reported by only a single facilitator.

Eggs were rarely given to the children at the ECD centres, which was reported by only one facilitator who mentioned that they sometimes give the children scrambled eggs when they have the resources. All the facilitators reported that they always prepare vegetables for the children. Additionally, for the food groups of vitamin A-rich fruits and vegetables and other fruits and vegetables, the only vegetables which the facilitators mentioned were spinach, cabbage, and carrots.

### 3.3. The State of Food Security at the Centres

Although the facilitators highlighted that they followed the menu in preparing the food for the children, they also reported that they usually substitute some of the foods with those available at the time. Most often, the money to buy food may not be enough for the types of food on the menu so they resort to cheaper ingredients with the same “nutritional value”. Thus, when certain foods are unavailable, meals are adjusted. For example, they reported that instead of preparing lean beef mince, they can substitute it with Imana soya mince soup or supply children with rice and cabbage instead of meat. In cases like these, all the facilitators highlighted that they do not worry about the nutritional quality of the substitute food as they perceived that the children will still consume the required nutrients for their growth and development. However, this was different from the observations that were made by the researcher during lunch times at three ECD centres, where children consumed rice with potatoes as their meal. About 50% of the ECD facilitators never worried about not having enough food to give the children, with 25% rarely worrying, whilst 12.5% of the facilitators worried sometimes and another 12.5% often worried that the children might not have enough food for the month (Table 2). Although these facilitators worried about not having enough food for the children, about 75% of the centres did not have any other sources to boost their food reserves. Only 25% rarely received support from Child Welfare and or Methodist Church, but their budget still did not always last the full month. However, these facilitators have reported that this has been disturbed by the COVID-19 pandemic and most of the support has since been stopped. Some of the centres receive additional support from doing raffles and other fundraising activities, as reported by 12.5% of the facilitators. None of the ECD centres had flourishing gardens, as observed by the researcher, although about 12.5% of the facilitators reported sometimes getting vegetables from their ECD gardens, with 25% rarely harvesting from their gardens.

The ECD centres mostly relied on the income that comes from the monthly school fees paid by the parents and from government subsidy. However, the government funds are disbursed quarterly, leading to financial strain on the ECD centres, especially in August and September, when funds are delayed. The subsidy usually arrives late, and not all parents pay the fees on time, thus leaving the ECD centres in a dire situation, with the facilitators mostly affected by the situation. When there is not enough food at the ECD centres, the facilitators may ask the parents to donate food to the centre, either from their homes or through purchased contributions and those who would not have paid school fees to do so. However, the facilitators reported that parents’ engagement varies. That is, not all parents get involved in the running of the ECD centres, as only 25% of the facilitators were positive about parental involvement. The other 75% highlighted that parents do not even attend the meetings they call to discuss such issues, even the strategic planning for the centres and to know what their children learn, eat, and use when at the centre. All the facilitators with this challenge often contribute personally to their ECD centres and cook meals to ensure all children are fed, whilst sharing the food equally amongst the children. Thus, they sometimes forgo their salaries and also for the support staff, which they can get reimbursed when they receive the government subsidy.

Sometimes when resources are limited, other children from the poorest homes who come to school with no food involuntarily consume limited variety and quantities of food. This was reported by 37.5% of the facilitators, although this happens rarely with 12.5% of these facilitators. For some of these facilitators facing the challenge of reducing the number of meals, porridge becomes the main meal. This becomes a bad experience for them as the children would ask questions relating to why they would not be having all the meals. For some facilitators, they skip porridge and only prepare lunch, which also hurts the children who are used to having porridge as their first meal the moment they arrive at school. One of the facilitators (Facilitator 1) pointed out in an interview that, “*sometimes they miss having porridge which really hurt. If the porridge is not available, they always ask why we are not having it and that is not a good thing to experience as a facilitator*”. Another (Facilitator 2) also said “*sometimes they (meaning children) do not have breakfast because of no resources, gas or if we do not have electricity to cook*”.

### 3.4. Perceptions of Food, Nutrition, and Health of the Children at the Centres

During the FGDs, all the facilitators highlighted that the food they provide to the children at the ECD centres is safe and nutritious, although they acknowledged that it is not enough for the children. They were confident with the children’s diet and nutritional intake, as they have reported that they receive support from the Department of Health who have provided them with balanced menus, guidance, and training on preparing nutritious meals for children. All the facilitators reported that they receive regular visits from the Department of Health personnel and dietitians, and they also do regular workshops to educate them on healthy meal preparation, food safety, and appropriate serving portions for children as well as appropriate snacks to give to the children. The facilitators also reported that the food they give to the children was safe, based on what they learnt about food safety from the health workers. To ensure food safety, they added that they always check expiry dates when purchasing the food. Similarly, all the facilitators were very confident with the meals they prepare for the children because the menu was planned by the dieticians, and they were taught and trained on how to cook the food and to maintain hygienic preparation conditions. They all agreed that healthy meals for children should be boiled not fried.

The facilitators also highlighted the nutritional value of meals, noting that the meals they prepare are well-balanced and they contain essential vitamins from vegetables, soya, and meat which support the children’s health and growth. They always make sure to include vegetables in every meal to increase the quality of the food (except when specifically having meals like umphokoqo (pap) and maas), thus improving the nutritional status of the children.

Facilitator 3 emphasised that “*Meals are considered healthy as long as they include vegetables, whether cooked or fresh*”. Furthermore, all facilitators agreed on providing children with a balanced diet which has “nutritional variety” so that they may become healthy. Another facilitator (Facilitator 4) noted that “*balanced and healthy meals should contain a mix of vegetables, protein (meat, lentils, cheese, yoghurt) and staple foods like rice and mealie meal*”. However, they face some challenges with children’s eating habits. Despite efforts to include vegetables in the meals, most facilitators reported that children at their centres often refuse to eat them, although they are essential in children’s meals.

The ECD facilitators also believed that meal structure and frequency of eating are very important factors for children’s health and growth. For example, in the FGDs, it was agreed by all facilitators that a nutritious diet for children includes porridge as the first meal, a variety of daily fruit and water intake, and a full meal with vegetables, always. However, although they all had this extensive knowledge of the IYCF practices, they all admitted that there were challenges in implementing these diets, with certain adaptations needed. For example, they highlighted that due to financial constraints faced by parents and the ECD centres, most children often receive the same type of fruit such as an apple, with some not bringing any fruit from home. Also, dietary adjustments are sometimes made for meat with Imana soya mince soup or potatoes and also for picky eaters, such as substituting umphokoqo and maas with noodles. Another facilitator (Facilitator 3) spoke about these adjustments she makes saying “*sometimes, if there is no meat I mix rice with cabbage*”, which all the other facilitators in the FGD agreed with, while others mentioned substituting meat with potatoes as another form of adaptation. Similarly, Facilitator 5 also agreed to this, saying “*we do not usually follow the menu every time because we do not always have the things on the menu, so we improvise. We sometimes cook lentils, mincemeat, or if no meat we use soup with soya (Imana), if no matabela we cook maize porridge or oats. Weet-bix are very expensive and require a lot of milk which is also expensive*”.

With regard to the health of children at the ECD centres, all facilitators agreed that the programme run by the Department of Health helps them to keep the children safe from malnutrition. The health professionals conduct child nutrition checks, provide vitamins, and assess meal plans every month at their centres. The ECD facilitators also received training on measuring the weight, height, and mid-upper arm circumference of the children to detect the children’s nutritional status. Sadly, and as reported by all the facilitators, some children rely entirely on the ECD centres for food and their health declines during the holidays due to lack of proper meals at home. Facilitator 6 commented on this, saying “*we do weighing of children and measure MUAC every month to determine health of the children. Kids’ health mostly deteriorates during holidays as they only eat porridge (ACE instant) at home. Parents don’t make sure that their children eat vegetables and fruit*”. All the facilitators have agreed that they notice negative changes in some of the children’s health, mostly after the holidays, which they report to the dieticians who then advices them to add more peanut butter in those children’s food. Therefore, the children’s diets are supplemented with peanut butter when signs of malnutrition appear. Also, the facilitators have noted the lack of variety in fruits and proteins due to cost constraints, which limits the dietary diversity of the foods consumed by the children. One of the facilitators (Facilitator 4) said “*there are children who do not grow but there is only a few of them. We report to the dieticians. The dietician then advises us to put more peanut butter in the food*”, whilst Facilitator 8 agreed, saying “*some children at the ECD are not growing as is expected and in this case I report to the Department of health/dieticians where they tell me to feed the children with more peanut butter. When I do this, I see change but I do not know if the children are also getting help from home*”.

In situations like these, all the facilitators highlighted that they also inform the parents in a proper way about their children being malnourished as this is a sensitive issue, but some parents do not receive it well; they remain in denial mode and insist that their children are well. These parents are said to not act on the situation and the children may not receive medical help, as noted by the facilitators, and therefore the facilitators may not be able to do anything for the children because they also fear victimisation, hence maybe choosing their personal safety and life. However, other parents accept the situation and may seek help from the Department of Health and work with the facilitators who would work closely with the dieticians and nurses consistently, following the dietary guidance when feeding the children at school and home until the children’s health improves. Facilitator 7 positively reflected on this process, saying “*Learning and doing a course on how to treat children as a facilitator is very important as it helps you to be able to take care of them (meaning children). It motivates me and gives me strength to continue doing my job smoothly seeing the health of children improving*”.

Additionally, a few parents were said to have been engaged in the ECD centres’ nutrition programme through providing food such as potatoes, rice, and butternut at the centres. Thus, one parent would bring a single item to replenish the centre’s food basket when it is dry, while some parents actively contribute snacks and toiletries. The facilitators mentioned that these few parents were also present in the consultation meetings at the ECD centres to introduce the new menu. They were encouraged to follow healthier cooking methods at home such as “boiling” without overcooking instead of “frying” the food and always ensuring balanced portions for their children. Additionally, the facilitators who had mentioned having vegetable gardens at their centres also reported that, although other parents may be less involved in the ECD centres’ activities, including maintaining the school garden, a few parents, especially those from disadvantaged backgrounds, assist them with gardening, thus learning skills which they can apply at home, and may also receive vegetables in return. Unfortunately, access to water has been reported to be a challenge at these centres, mainly due to stolen taps and pipes, thus impacting ongoing gardening projects. Therefore, although food gardening has helped to provide food and reduced costs at the ECD centres, the facilitators reported that continuation was limited due to infrastructure constraints as well as limited access to reasonable space (land). Although the majority of the parents and caregivers may not be aware of the situation at the ECD centres, including the challenges, since all the responsibilities are left for the facilitators to resolve, the facilitators have reported that they always try by all means to communicate the situation with parents.

### 3.5. Possible Solutions to the Challenges Faced by ECD Centres in Makhanda

During the FGDs, the researcher and the facilitators further discussed about the possible solutions that can be implemented to improve the food insecurity status at the ECD centres, especially when government subsidy arrives late and when some parents are not able to pay the fees. Three main thematic areas emerged from the discussions and these themes highlighted practical and collaborative approaches to sustaining ECD centres despite all the challenges they face, including financial constraints. The thematic areas included the following:

#### 3.5.1. Sustainable Financial Solutions for Delayed Government Funding and Fee Payments

All the ECD facilitators agreed that there is a need for strategic financial planning to minimise the challenges they face when government funding is delayed and when some parents have not paid school fees. This included drafting a budget that allows them to save surplus funds from previous payments which they may use to cover expenses during delays. The planning may also include fundraising initiatives, where they may organise some fundraising activities at the ECD centres, with support from local businesses through small monthly donations of funds or supplies. Some of the fundraising activities suggested included selling homemade cupcakes to children every Friday or implementing talent shows where they may charge an entry fee and sell food and items donated from local businesses. The funds raised may then be used to buy some fortified supplements such as other nutrient-dense porridge or more peanut butter including peanut butter paste which can be given to children in need of nutrient supplementation. However, most facilitators have at least once tried raffle fundraising, which they all agreed was not successful, with very few children having sold tickets. They also agreed that parental contributions may lighten the burden on the ECD centres, especially if parents are encouraged to provide non-monetary support like food or toiletries to reduce financial strain. Some of the solutions were beyond the facilitators’ control, such as having an alternative salary payment structure for the facilitators and ECD centre support staff, where the government salaries are separated from the subsidy funds and come directly from the government to allow consistent staff payment. Similarly, the facilitators also highlighted the need for better government payment scheduling, also advocating for timely payments, ideally on the first day of each month rather than delayed quarterly payments.

#### 3.5.2. Food Gardens as a Sustainable Food Source

All the ECD facilitators in the FGDs agreed that one of the sustainable solutions to food insecurity at their centres is to own a flourishing food garden. They all acknowledged the benefit of food cost reduction through practising food gardening at the centres. Therefore, they highlighted the need to keep encouraging each other to establish food gardens which may help in lowering grocery costs, especially vegetables rich in essential nutrients like vitamin A such as spinach, which are very expensive to always purchase. However, for this to be successful, all the facilitators reported that they needed to gain some gardening training and attend some workshops, to which they may also invite parents so that they may also improve their gardening skills. Parental involvement in gardening projects at the ECD centres was supported by all the facilitators, where parents can assist in planting and take turns in maintaining the gardens at the ECD centre. They also highlighted that these skills may be transferred home, where parents may establish their own vegetable gardens. However, some facilitators hinted that this could not be viable considering the challenges with water, security, and very little or no gardening space at some of the ECD centres, as well as most parents being working class. The facilitators then agreed that in such instances, alternative methods to gardening may be employed such as use of tyres, basins, or small containers and tower gardens, which have been deemed successful methods for gardening in other areas with limited spaces. The facilitators acknowledged that this could be a way to allow flexibility in rotating different types of vegetables given to the children as needed. However, as they had mentioned that most children do not prefer vegetables, they noted that they may encourage them to eat vegetables through positive reinforcement and using creative methods to make vegetables appealing, including playful competitions such as whoever finishes vegetables first would be rewarded extra playtime. They all admitted that this was a way to encourage healthy eating in children and that with time, all the children would adapt to eating vegetables, even as a raw vegetable snack.

Unfortunately, the issue of access to water remains a challenge to proper gardening and has been an unresolved issue in the discussions, as this has become a town-wide challenge. Therefore, the discussions on gardening shifted from having it on the ECD centres to either partnering with local NGOs and local governments and Rhodes University for access to resources like land; seeds, including drought-resistant seeds; technical support; and funding, with the ECD centres joining forces to create a “community food bank” where surplus produced can be stored and shared amongst the ECD centres involved in the programmes. The facilitators further concurred that the surplus produce (including seedlings) can even be sold to sustain the food programme and engage local communities in the formation of a micro-food enterprise for the ECD centres in Makhanda. With these suggestions, the research team then involved the Rhodes University’s Grounds and Gardens and the Division of Student Services and Development in the discussions to collaborate in these gardening initiatives. It was also agreed that these interventions needed to be performed practically in a more pragmatic way, where ECD facilitators would be fully supported, especially in the ECD centres that were experiencing severe food insecurity. Additionally, everyone acknowledged that the ECD facilitators needed to be involved in the intervention programmes and decision-making, and later pull in parents, in order for the initiatives to succeed. Through working together, the impacts of malnutrition in Makhanda’s young children may be reduced.

#### 3.5.3. Increasing Parental Involvement in Children’s Nutrition

The challenge faced by all the facilitators of taking on all the responsibilities of the children’s nutrition and health sparked a discussion where possible solutions to strengthen parental involvement in children’s nutrition and health were agreed upon. The facilitators agreed that they cannot fully improve the children’s health at the ECD centres alone without the help and consistent involvement of parents or the child’s caregivers. To strengthen parental involvement at the ECD centres, some facilitators suggested that one of the viable solutions would be to implement workshops for parents to educate them on IYCF practices and the food needs of the children (as learnt from the dieticians), as well as advising them on the challenges the facilitators face and advocating for collaborations. All the facilitators concurred with this suggestion. Within these workshops, the facilitators highlighted that they may also introduce the need for shared gardening initiatives to allow parents to contribute fresh produce to the ECD centres’ food baskets which may span to their households. All the facilitators noted that for this to be successful, there is a need to maintain open communication with parents to share progress, updates, and challenges through WhatsApp groups and monthly ECD newsletters, to ensure that parents may stay informed about nutrition and health situation at the centres. They suggested that these channels can also be used to share nutrition information with parents. Hence, all the facilitators acknowledged that parents need to be engaged earlier from the beginning of the year to allow for continuous engagement, rather than only when there is a crisis at the ECD centres. The facilitators noted that this may help with strengthening their collaborations with parents through creating a proactive approach to children’s nutrition, health, and wellbeing, rather than a reactive approach only in times crisis.

Noticing health issues in some children and facing parental denial was one of the key challenges and a major concern which warranted the need for parental involvement, as pointed out by all the facilitators. This was usually noticed after the school holidays, during which the facilitators believed the poor health of children often went unnoticed or unacknowledged by their parents and therefore, inviting parents to educational sessions/workshops carried out by health professionals might address the issue. All the facilitators believed that parents needed to hear this information directly from the health professionals, which might encourage consistent healthy eating habits at home. Thus, they perceived that professional health workers’, especially dieticians’, nutrition sessions and training on how to identify malnutrition at home may help parents understand the importance of nutrition and the positive results of providing healthy nutritious meals to children and how unhealthy menus may lead to malnutrition. The facilitators highlighted that this might also help with avoiding straining relationships with the parents when their children are noticed as suffering from malnutrition. However, some of the facilitators pointed out that the illnesses observed in some children were not only caused by poor diets or not having enough food to eat, but from allergies and infections, which parents might also be made able to distinguish. Additionally, they all agreed that engaging the children in discussions about nutritious food choices may be beneficial as this can influence their parents’ food choices and consumption behaviour and can bring them closer to the centres, as their children may also pass the nutritional and healthy eating information home. Some facilitators had also suggested having ECD food policies, especially having restrictions on certain foods that children bring to school. However, this suggestion yielded mixed feelings in the FGD meeting as some facilitators pointed out that the financial constraints that a number of parents face mean that they may not be able to always pack healthy foods such as fruit for their children. While the researcher had asked if it was a viable solution for the ECD centres to provide food to the children during holidays to avoid poor health post-holidays, all the facilitators disagreed and had mixed opinions to this. Some facilitators pointed out that it could be challenging due to financial constraints and the logistic to do so as they also need rest and personal time with their families during the holidays. Others also advocated for social workers to organise soup kitchens within the communities, where children can go and receive a meal instead. However, in so doing, they all acknowledged and believed that it is the parents’ responsibility to make sure that their children are well fed with nutritious meals all the time.

## 4. Discussion

Adequate and diverse diets are essential for children’s physical and cognitive development, yet food insecurity and malnutrition remain critical challenges in resource-poor settings. The findings of this study have shown that the ECD centres in Makhanda face financial constraints which severely impact the quality of care provided to children, children’s nutrition, and sustainability. The ECD centres rely on inconsistent government subsidies and parental fees, leading to food shortages for the children. This further exacerbates the burden on facilitators, who already face delayed salaries and are forced to personally contribute food and resources, thus highlighting job insecurities for the ECD centres staff. These findings align with other research studies on ECD centres, where financial and resource constraints faced by ECD centres have been reported to threaten the nutrition and dietary patterns of young children, leading to reliance on parental contributions from fee payment [49,50]. Furthermore, studies by Black et al. [51] and UNICEF [52] have noted that nutrition during the first 1000 days of life is critical for cognitive and physical development, yet many ECD centres struggle to provide adequate and nutritious meals due to limited funding. The report revealed the sad reality of malnutrition, where 149 million children under 5 years are stunted and almost 50 million are wasted, while overweight and obesity in children is continuing to rise, increasingly among the poor due to healthy diets becoming more expensive while unhealthy, non-nutritious diets are becoming cheaper and affordable. Additionally, the present study found that the average amount spent on food per child per month at the ECD centres in Makhanda was R90 ± R25 which concurs with reports on underfunding in ECD settings leading to insufficient food provisions to children [49] and thereby increasing food insecurity at the ECD centres. This is also exacerbated by financial instability, partly due to non-payment of school fees by parents, which forces the ECD facilitators to cut food costs, often through substitutions of expensive food items for cheaper ones. Thus, many ECD centres, especially in resource-poor settings, rely on cost-effective but less diverse diets to meet children’s nutritional needs, which has also been highlighted in studies by Vorster et al. [53], who noted nutritional transitions affecting many people in low-income countries.

Vorster et al. [54] has also advocated for food dietary guidelines for children in South Africa to change the eating behaviour towards more optimal diets that meet energy and nutrient requirements. However, the ECD facilitators in the present study were confident that the meals provided at the ECD centres were nutritious because they followed dietary guidelines, using menus and guidance received from dietitians, although, in reality, the food provided at the centres was often insufficient, leading to modifications and substitutions based on available resources. This is consistent with the report by Swart et al. [55], who noted that financial constraints often result in compromised dietary diversity for young children, although ECD centres generally strive to provide balanced meals. Like the first 1000 days, the next 1000 days are also critical in a child’s health and development. Therefore, governments need to necessitate investments that build upon early life interventions, encompassing responsive caregiving and parenting programmes, health, nutrition, and early learning, which are essential for optimising children’s physical and cognitive growth during this stage [3,56]. To improve the developmental outcomes of children both in the first and the next 1000 days, a multisectoral approach integrating health, nutrition, and education, as well as social protection in the form of funding, is vital. Additionally, strengthening policies and increasing community-based initiatives as well as investing in ECD research can help bridge the nutritional and financial gaps in the ECD sector. Similarly, ensuring the continuity of nurturing care across the home, the ECD centres and policy frameworks is essential for the long-term success and sustainability of the ECD centres [56], especially in resource-poor settings such as Makhanda.

The dietary patterns observed in the present study reflect both strengths and challenges in ensuring a diet which is balanced and nutritious for children enrolled at the ECD centre. This is highlighted by the provision of three meals per day at the centres, which is guided by Department of Health dieticians, thus demonstrating an effort to adhere to dietary guidelines for infants and young child feeding [10,18,44]. However, the facilitators have reported frequent substitutions of the unavailable food items to cheaper, less nutritious foods due to lack of funds and thus often prioritising cost of food over dietary diversity. The substitution of nutritious foods with cheaper alternatives, as reported in this study, is a common coping strategy among financially strained ECD centres. This aligns with other studies on household coping strategies to food insecurity, which similarly affect young children [57,58]. While substitutions such as replacing meat with soya mince soup or providing rice with cabbage may provide some nutrients, they may not adequately meet the dietary requirements necessary for optimal child development. This is concerning, especially with regard to long-term child health outcomes, given the evidence that dietary diversity is crucial for child development [17,59].

The study has shown heavy reliance on grains, roots, and tubers at the ECD centres, with limited intake of dairy, protein-rich foods, including eggs, and rare consumption of vitamin A-rich vegetables and fruit, which aligns with findings from other studies on child nutrition in low-resource settings, where affordability dictates food choices [9,17,60,61,62]. Furthermore, the observation that children in the study sites often bring processed foods like chips and noodles for their mid-morning snack instead of fruit also highlights the growing concern of unhealthy eating habits in young children. This challenge has been previously noted in other studies by Chakona [9] and Chakona and Shackleton [17], who observed poor nutritional quality diets and inappropriate early child feeding practices which are exacerbated by a greater access to processed foods. Although the facilitators have shown signs of resilience and attempted to compensate for dietary deficiencies in the children who do not bring food to school by sharing food and encouraging acts of kindness, without additional financial, community/stakeholder, and parental support, this practice is not sustainable in the long-term. To enhance resilience in ECD settings in Makhanda, this study highlights key strategies such as improved financial planning, promoting structured nutrition programmes including food gardens, and increased parental involvement as crucial for sustainable improvements. Diversified funding, parental nutrition education, and stronger community partnerships are also needed to increase collaborations to help mitigate financial gaps and food insecurity challenges faced by ECD centres. Also, improving the policy to ensure timely government funding to the ECD centres is crucial.

While some facilitators in the present study did not worry about food shortages, others frequently expressed concerns, which is consistent with literature highlighting the precarious nature of food access in South Africa [60,62,63], especially for young children [9,17,63,64]. Several studies have also shown that food insecurity in early childhood is linked to stunted growth [17,51,63], which may lead to irreversible changes in child development including poor cognitive development and increased susceptibility to infections [51,63]. The lack of flourishing food gardens at the ECD centres and their surrounding communities further exacerbate the food insecurity situation at the centres. Despite reported efforts to establish gardens at the centres, resource shortages, security issues, and infrastructure challenges have been hindering these efforts. Notwithstanding the COVID-19 pandemic, its impacts were also devastating at the ECD centres in the present study, as was also reported by Hendriks [65] in many communities in South Africa. However, it has been noted that school gardens can serve as a sustainable solution to food insecurity with producing fresh produce for the children whilst fighting against diet-related diseases and educating them about healthy eating habits [66]. The increasing recognition of the right to adequate food has strengthened the global commitment to addressing child malnutrition, thus making increased investment in nutrition both an economic necessity and a moral imperative [56,67], which may bring high human and economic returns in the long-term [68]. Therefore, promoting dietary diversification through establishing nutrition-sensitive gardens at the ECD centres with emphasis on food production and trainings to care for and keeping gardens productive. This may increase access to diversified nutrient-rich foods, which is crucial for achieving sustainable development goals, especially SDG 2 (zero hunger), SDG 3 (good health and wellbeing), and SDG 4 (quality education), which concurs with FAO [66]. Although the present study has shown the failure of gardening initiatives at the ECD centres, mostly due to limited resources and space, there is a need for encouragement and raising awareness of the importance and benefits of food gardens through nutrition workshops.

Nutrition education integrated with community empowerment and gardening skills training programmes can also improve communities and families’ nutrition behaviour and hence influence IYCF practices [23,24]. However, these initiatives should enable the ECD facilitators and surrounding communities to actively participate in decision-making, allowing for an inclusive and well-designed nutrition education, behaviour change approaches, and community empowerment approaches targeting mothers and caregivers. A study by FAO [30] has also suggested that allowing multidisciplinary/intersectoral collaborations may translate into improved diets and nutrition for children. The empowerment of local communities, including mothers and ECD facilitators, through agricultural practices and nutrition education may promote access to affordable quality diets. Furthermore, the knowledge gained enhances local capacities, with many households and community members being able to feed themselves and their infants and young children with diversified diets which have high potential to improve their nutritional status [23,24,27,28,29]. Therefore, a mix of nutrition education and child-centred nutrition-sensitive agriculture through gardening projects can be a powerful intervention strategy to fight infant and young child malnutrition in South Africa, especially if tailor-made and contextualised for Makhanda, where there are high rates of child stunting [32,38].

Parental involvement in the ECD centres’ functioning remains a critical factor in ensuring their sustainability and strengthening healthy eating habits, both at school and at home. The study observed that parental engagement in the ECD centre operations, such as contributing food or providing financial support for child nutrition as needed, remains a significant challenge, further exacerbating food insecurity at the ECD centres. This is evidenced in the present study through the non-payment of fees by parents, failure to attend ECD meetings, inconsistent food provisions from home, and parental denial to work with ECD facilitators and dieticians when a child has been noticed to be malnourished. Additionally, the reports of some children not bringing food from home, or bringing unhealthy foods, coupled with need for facilitators to provide nutritious foods in difficult circumstances, suggest that some parents may lack adequate knowledge of IYCF practices and may not completely recognise the importance of consistent nutrition provision in child health and development. Doherty et al. [25] has noted that one determinant factor of child health is promoting nutrition education for mothers and caregivers with young children (≤5 years) and findings by Shisana et al. [15] also showed that parental involvement in ECD nutrition programmes significantly improves children’s dietary habits and overall health outcomes. Similarly, Draper et al. [3] and UNICEF [69] have also emphasised the need for parental education on nutrition, particularly in low-income communities, to bridge knowledge gaps and ensure that children receive regular meals with balanced nutritional intake and dietary adequacy both at home and school. Most importantly, knowledge of appropriate child feeding practices is of fundamental importance for the survival, growth, development, health, and nutrition of infants and young children [37] and therefore it is crucial for parents and caregivers to increase their nutritional knowledge through participating in nutrition education programmes. Maternal and caregiver education have also been highlighted to have significant impacts on reducing child malnutrition [35,36], which has long-term human and economic benefits, as noted by Development Initiatives [68].

Initiatives such as participating in nutrition workshops and food preparation trainings conducted by the Department of Health dieticians, as well as using digital platforms like WhatsApp to communicate with parents, as suggested in this study, have also been recommended in other studies as effective strategies to enhance parental involvement [70]. Additionally, social media has been widely accepted as a popular platform for communication globally and has been used for learning in many communities in South Africa, especially in promoting sustainable agricultural practices in rural areas [71]. Transparent communication between ECD facilitators and parents through social media is significant in fostering stronger relationships, which can increase parental involvement in ECD programmes, especially among parents with limited educational attainment, as observed by Fatema and Lariscy [72], who might be shy to come out publicly. Additionally, ECD facilitators have also suggested integrating nutrition discussions into ECD activities to educate children and influence their food preferences, thus indirectly educating the parents. The approach is also supported by FAO [66] who suggests needs-based learning with practical, real-life aims for children to develop their capacities to adapt to, promote, and drive change, particularly on the types of food they consume, thus developing essential food competences.

Thus, ECD centres provide platforms for nutrition intervention, offering opportunities for learning, infant and young child feeding, and health screenings, although there is urgent need for creating platforms for parental education, as observed in the present study. The findings concur with Draper et al. [3], who noted that early childhood care and education settings provide promising platforms for interventions and offers opportunities for parental education, school feeding, and health screening, which complement ECD services and thus foster improved growth and development in children. Other studies have also reported that parental educational programmes [34,35,36,37] and integrating health workers into school programmes yields major improvements in health behaviour and outcomes in children [73]. This may yield sizeable improvements in children’s developmental outcomes at the ECD centres [56], especially in Makhanda. However, Hui et al. [33] and Vasilevski and Carolan-Olah [36] noted that for intervention programmes that promote optimal feeding practices for the infants and young children to be successful, there is a need for commitment by all stakeholders, including parents. The ECD facilitators also justifiably highlighted the ultimate responsibility of parents in ensuring children’s nutrition at home both during holidays and the school term, thus strengthening the need for family participation in IYCF intervention programmes. Similarly, the suggestions for establishing soup kitchens in Makhanda as an alternative ECD-based feeding programme during holidays shows the need for community-driven solutions to food insecurity and bridging nutritional gaps for children from food-insecure households, which agrees with Breckwich Vásquez et al. [74].

## 5. Conclusions

In conclusion, the study highlights the importance of a community-based participatory research approach in addressing food insecurity and malnutrition in ECD centres in Makhanda. The ECD facilitators were engaged as co-researchers, enabling the co-identification of challenges at the ECD centres and they participated in discussions on the development of localised, practical solutions, leading to a more relevant and effective interventions programme to be implemented in the town. Unlike traditional top-down models, CBPR as a valuable engagement strategy enhances the sustainability of the ECD centres using context-specific interventions such as food gardening, food banks with vitamin A-rich produce, financial literacy training, and sustainable ways of increasing parental involvement, while boosting community development. The study highlights the value of empowering facilitators/caregivers and local institutions ineffectively addressing food insecurity at ECD centres, particularly in settings facing unreliable government subsidies and low parental involvement with regard to fee payments and infrastructure limitations, which continue to obstruct progress. Although the ECD facilitators have adopted practical solutions such as dietary adjustments for children and continuous feeding for malnourished children, the challenges continue to exist, especially with limited engagement and reluctance posed by some parents to acknowledge childhood malnutrition. This reflects broader social and educational gaps that must be addressed through targeted awareness campaigns within the Makhanda community. Thus, the study reinforces the importance of linking nutrition education with local food systems and collaborative policy engagement, where education initiatives should target parents, equipping them with knowledge about childhood nutrition and the importance of early dietary interventions. Strengthened government support, timely subsidy disbursement, promoting financial diversification, and integrating policy reforms specific to nutrition budgeting and early childhood subsidies are needed to enhance the resilience of ECD centres. Expanding local food production and having food banks for the ECD centres, engaging fundraising activities to generate financial support, and promoting financial literacy among ECD staff enhances resilience and sustainability in ECD nutrition programming across resource-constrained communities. Future research should expand on this approach to develop scalable models that leverage local capacities, local food systems, community partnerships, and align with policy frameworks to create a more sustainable and inclusive approach to ECD nutrition, where every child’s right to nutritious, diverse, and sustainable food is upheld in South Africa and beyond.

## Figures and Tables

**Table 1:** Sample distribution by ECD centres and child demographic characteristics.

	Indicator	Category	% (*n* = 307)	% of Girls per Centre	% of Boys per Centre
Early Childhood Development Centres’ characteristics	Early Childhood Development Centres	ECD 1	15	56.5	43.5
		ECD 2	8.5	57.7	42.3
		ECD 3	13	52.5	47.5
		ECD 4	13.7	26.2	73.8
		ECD 5	8.8	29.6	70.4
		ECD 6	19.5	56.7	43.3
		ECD 7	11.4	45.7	54.3
		ECD 8	10.1	48.4	51.6
	No. of children	Range		26–60	
		Mean no. of children per centre		38 ± 11	
	ECD food expenditure in Rand per month	Range	R1300–R5000 *		
		Mean ECD centre food expenditure	R3563 ± R1489 *		
	Food expenditure in Rand per child per month	Range	R50–R119 *		
		Mean food expenditure per child	R90 ± R25 *		
	Mean time spend at school in hrs	8.1 ± 1.4			
Children’s characteristics	Gender distribution at the centres	Mean no. of girls	18 ± 8		
		Mean no. of boys	20 ± 6		
		% of girls (*n* = 146)	47.6		
		% of boys (*n* = 161)	52.4		
	Age of children in months	Range	7–60 months		
		Mean age of children per centre	36.8 ± 21.5		
		Mean age of youngest child per centre	16.6 ± 6.3		
		Mean age oldest child per centre	57 ± 4.5		

* $ 1 (USD) was approximately R19 (ZAR) at the time of study.

**Table 2:** The state of food security at ECD centres and other factors in their positive functioning.

Percentage of ECD Facilitators and Times They Worry per Month				
Variable	Never	Rarely (1–2 Times)	Sometimes (3–10 Times)	Often (>10 Times)
ECD getting vegetables from gardening	62.5	25	12.5	-
ECD centres receiving donations	75	25	-	-
ECD centres doing fundraising	87.5	-	12.5	-
Worry about children not having enough food	50	25	12.5	12.5
Times when children receive fewer meals	62.5	12.5	25	-
Parental involvement	-	25	50	25

## Data Availability

The data presented in this study are available on request from the corresponding author and after obtaining permission to share the data from the study’s participants.

## Abbreviations

The following abbreviations are used in this manuscript:

CBPR	Community-Based Participatory Research
CSD	Centre for Social Development
DBE	Department of Basic Education
ECD	Early Childhood Development
FAO	Food and Agriculture Organisation
FGDs	Focus Group Discussions
IYCF	Infants and Young Child Feeding
MUAC	Mid-Upper Arm Circumference
NDoH	National Department of Health
NIECD	National Integrated Early Childhood Development
SADHS	South Africa Demographic and Health Survey
SANHANES	South African National Health and Nutrition Examination Survey
SDGs	Sustainable Development Goals
Stats SA	Statistics South Africa
UNICEF	United Nations Children’s Fund
WHO	World Health Organisation
